# Reconstruction of the genome-scale metabolic network model of *Sinorhizobium fredii* CCBAU45436 for free-living and symbiotic states

**DOI:** 10.3389/fbioe.2024.1377334

**Published:** 2024-03-25

**Authors:** Anqiang Ye, Jian-Ning Shen, Yong Li, Xiang Lian, Bin-Guang Ma, Feng-Biao Guo

**Affiliations:** ^1^ Department of Respiratory and Critical Care Medicine, Zhongnan Hospital of Wuhan University, School of Pharmaceutical Sciences, Wuhan University, Wuhan, China; ^2^ Key Laboratory of Combinatorial Biosynthesis and Drug Discovery, Ministry of Education, Wuhan University, Wuhan, China; ^3^ Hubei Key Laboratory of Agricultural Bioinformatics, College of Informatics, State Key Laboratory of Agricultural Microbiology, Huazhong Agricultural University, Wuhan, China

**Keywords:** genome-scale metabolic models (GEMSs), *Sinorhizobium fredii* CCBAU45436, biolog phenotype microarray, proteome data, essential genes, fixed NH_3_

## Abstract

*Sinorhizobium fredii* CCBAU45436 is an excellent rhizobium that plays an important role in agricultural production. However, there still needs more comprehensive understanding of the metabolic system of *S*. *fredii* CCBAU45436, which hinders its application in agriculture. Therefore, based on the first-generation metabolic model *i*CC541 we developed a new genome-scale metabolic model *i*AQY970, which contains 970 genes, 1,052 reactions, 942 metabolites and is scored 89% in the MEMOTE test. Cell growth phenotype predicted by *i*AQY970 is 81.7% consistent with the experimental data. The results of mapping the proteome data under free-living and symbiosis conditions to the model showed that the biomass production rate in the logarithmic phase was faster than that in the stable phase, and the nitrogen fixation efficiency of rhizobia parasitized in cultivated soybean was higher than that in wild-type soybean, which was consistent with the actual situation. In the symbiotic condition, there are 184 genes that would affect growth, of which 94 are essential; In the free-living condition, there are 143 genes that influence growth, of which 78 are essential. Among them, 86 of the 94 essential genes in the symbiotic condition were consistent with the prediction of *i*CC541, and 44 essential genes were confirmed by literature information; meanwhile, 30 genes were identified by DEG and 33 genes were identified by Geptop. In addition, we extracted four key nitrogen fixation modules from the model and predicted that sulfite reductase (EC 1.8.7.1) and nitrogenase (EC 1.18.6.1) as the target enzymes to enhance nitrogen fixation by MOMA, which provided a potential focus for strain optimization. Through the comprehensive metabolic model, we can better understand the metabolic capabilities of *S*. *fredii* CCBAU45436 and make full use of it in the future.

## 1 Introduction

Rhizobia are Gram-negative bacteria that can fix nitrogen from the air by parasitizing in plant nodules and transport it to host plants meanwhile the host provides carbon and other nutrients in return. Unlike rhizobia, free-living nitrogen-fixing bacteria such as *Azotobacter chroococcum* (Song et al., 2020) can autonomously fix nitrogen and have no dependency on the plant body. Among various biological nitrogen fixation systems, the legume-rhizobium symbiosis holds the highest efficiency and prominence. Symbiotic nitrogen fixation (SNF) by rhizobia is an effective way of biological nitrogen fixation, which is of great help to agricultural production (Yang et al., 2017; Lindstrom and Mousavi, 2020). To form SNF, rhizobia firstly need to recognize and produce specific signal molecules, which helps to form specialized tissue differed from host plants and finally become bacteroids (Timmers et al., 2000). By the symbiotic form, rhizobia release ammonium into the host plant in exchange for a supply of carbon and nutrients, which sustains a mutually beneficial relationship between plants and rhizobia (Prell and Poole, 2006). So far, a variety of rhizobia have been found like *Bradyrhizobium diazoefficiens* USDA110, *Sinorhizobium meliloti* 1,021 (Hwang et al., 2010), which have the potential to help increase production in agriculture, and *S*. *fredii* is a typical rhizobium widely used on alkaline-saline land (Han et al., 2009). *S*. *fredii* CCBAU45436 is the dominant strain among a dozen sub-lineages that can perform nitrogen fixation with some Chinese soybean cultivars efficiently (Munoz et al., 2016).

To better understand the metabolic capabilities of bacteria, genome-scale metabolic network models (GSMMs) are widely used. GSMMs are mathematical models that have become crucial systems biology tools guiding metabolic engineering (Ye et al., 2022). At present, the GSMMs of model organisms such as *Saccharomyces cerevisiae* (Heavner and Price, 2015) and *Escherichia coli* (Weaver et al., 2014) are relatively comprehensive, but there are still many blanks for non-model organisms, which results in significant limitation for their utilization. There are some GSMMs of none-model organisms like *i*ZM516 (Wu et al., 2023), *i*QY1018 (Yuan et al., 2023), and iZDZ767 (Zhang et al., 2023) which reveal potentially efficient ways to produce substances with economic benefits by microorganisms (Huang et al., 2023). In 2020, Contador and others developed *i*CC541, the first generation of the metabolic network model of *S*. *fredii* CCBAU45436 (Contador et al., 2020). However, as it contains inadequate genes and reactions, it is difficult to well reflect the whole metabolism of the rhizobium. On the one hand, it scored 70% in the latest version of the MEMOTE test, indicating its imperfect aspects, which need to be updated. In addition, it used old locus tag and EC number, which is inconvenient to understand and apply, so we urgently need a more complete metabolic network model to comprehensively reflect the metabolism of the rhizobium.

The reconstructed metabolic network model, *i*AQY970, is a powerful tool for studying nitrogen-fixing bacteria, which can well reflect its metabolic status. The reconstruction was based on *i*CC541, on which new genes and reactions were added from databases like KEGG, ModelSEED and MetaCyc in line with the newest annotation information. The gap-filling process helped to enhance the global connectivity of the model by reducing the gaps caused by imperfect annotation information. Biolog phenotype microarray can directly measure an organism’s physical performance in a specific environment (Shea et al., 2012). With the experiments, we can test the capacity of utilizing different carbon, nitrogen, sulfur sources and other nutrients of the bacteria, by which we can validate the simulation accuracy under the free-living condition of the model. It has been reported that the correlation between transcriptome and proteome data of nitrogen-fix bacteria was not strong (Rehman et al., 2019), so we used proteome data as the second data. Integrating proteome data into the model, we established models under different conditions, which was a good method to predict the metabolic status of the rhizobium in specific circumstances. We further validated the simulation of the metabolic model by comparing the predicted results with experiment information published in previous literature. Finally, we predicted four possible modules and two target genes in *i*AQY970 with a great impact on the rhizobium during nitrogen-fixing period, which provided guidance to improve the efficiency of nitrogen fixation through strain modification in the future.

## 2 Materials and methods

### 2.1 Draft model for *S. fredii* CCBAU45436

**FIGURE 1:**
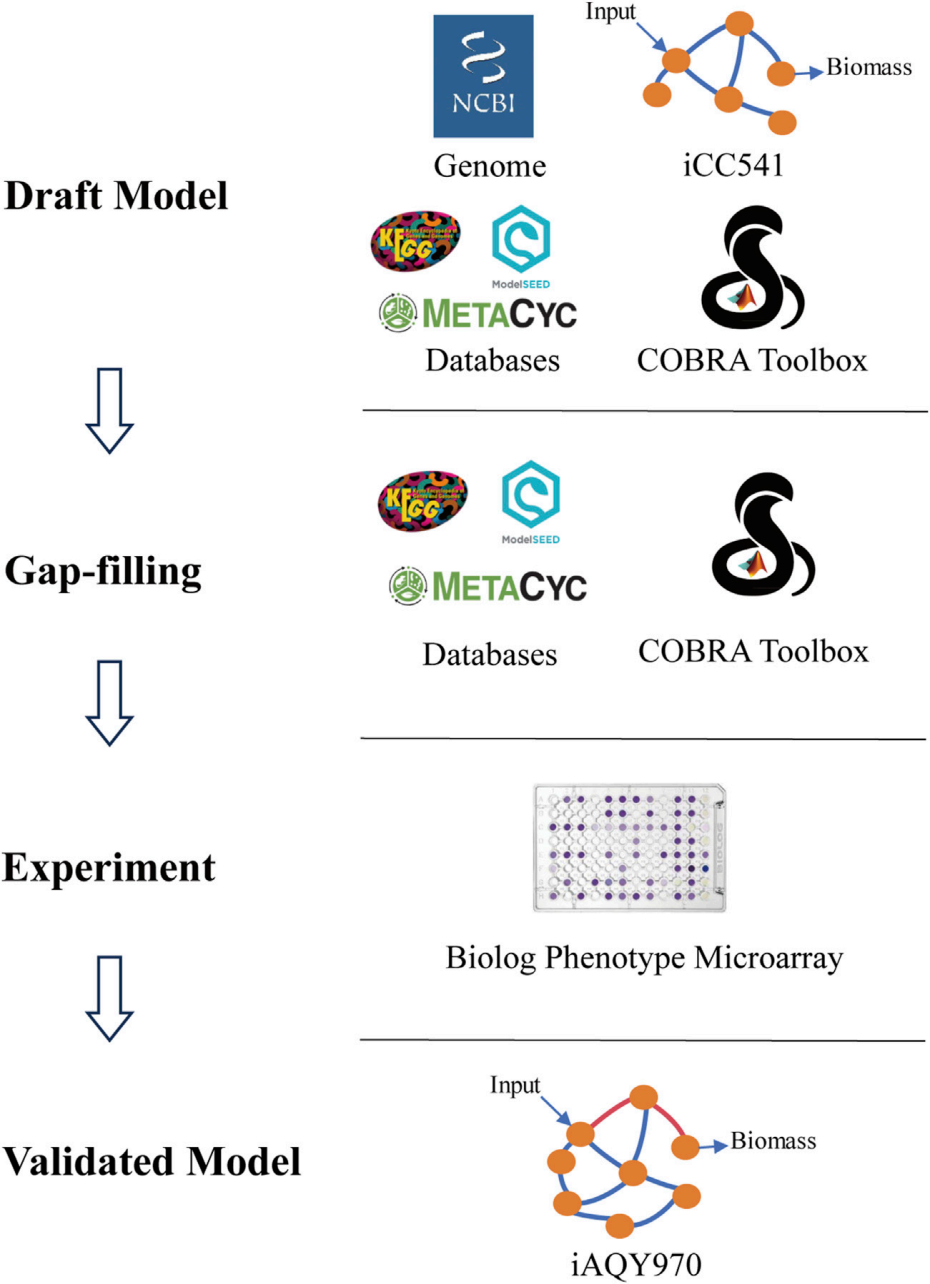
Workflow of metabolic network reconstruction *i*AQY970.

The new model was constructed by manual method following the protocol including draft reconstruction, refinement of reconstruction, conversion of reconstruction into computable format, network evaluation, and data assembly and dissemination (Thiele and Palsson, 2010) (Figure 1). The *i*CC541 model was used as a template, and the metabolic network was reconstructed by adding reactions from KEGG (https://www.kegg.jp/kegg/) (Kanehisa and Goto, 2000), ModelSEED (https://modelseed.org/) (Seaver et al., 2021), and MetaCyc (https://metacyc.org/) (Caspi et al., 2020) databases. The genome information used in the reconstruction process was downloaded from NCBI (https://www.ncbi.nlm.nih.gov/datasets/genome/GCF_003100575.1), and *i*CC541 model was obtained from the GitHub website (https://github.com/cacontad/SfrediiScripts). The annotation information of genes, metabolites and reactions corresponding to other databases in the model was based on the KEGG and ModelSEED numbers. SBO annotation was added according to MEMOTE test (Lieven et al., 2020).

### 2.2 Determination of the objective equation

The objective equation often determines the optimization direction of the entire metabolic model. In this study, the model was applied to the simulation of the rhizobium both in the free-living and symbiotic state, in which the biomass equation in the free-living state referred to the *i*GD1575 model (DiCenzo et al., 2016), and the symbiosis equation in the symbiotic state referred to the *i*CC541 model (Contador et al., 2020). They were modified according to the model construction. In the free-living state, the biomass equation includes DNA, RNA, proteins, phosphatidylethanolamine, poly-3-hydroxybutyrate (PHB), glycogen, and putrescine. The chemical formula of biomass can be found in Supplementary Table S1. To decrease the potential bias from flux balance analysis (FBA) (Chan et al., 2017), the molecular weight (MW) of biomass was set to be 1 g/mmol. In silico media compositions were 1.44 mmol g^−1^ h^−1^ malate, 1.38 mmol g^−1^ h^−1^ succinate, 1.26 mmol g^−1^ h^−1^ oxygen, 2 mmol g^−1^ h^−1^ glutamate and 0.01 mmol g^−1^ h^−1^ inositol, which were confirmed by previous literature (Contador et al., 2020).

### 2.3 Gap filling

Due to the inaccurate gene annotation information and reaction libraries, the draft metabolic network model often has gaps that damage the flux balance of the model. Based on the information of the KEGG pathway, we manually filled the gaps in the draft metabolic network model. There are four methods we used to reduce the gaps. Many metabolites have aliases that could cause them to be represented by different symbols in the model; we checked the metabolites of the model to ensure there were no duplicated metabolites in the model. By unifying duplicated metabolites, we lessened dead metabolites and improved connectivity. Also, the wrong direction of the reactions resulted in obstruction of the circulation of metabolites. So, we adjusted the direction of the reaction properly when it was wrong. However, if the methods above did not work, new reactions had to be added to connect metabolites. Some reactions became blocked for lack of sink reactions and demand reactions. The blocked reactions were checked by the function “findBlockedReaction” in COBRA Toolbox (Heirendt et al., 2019).

### 2.4 Biolog phenotype microarray experiment and analysis

Biolog phenotype microarray was a universal method to detect the absorption of different substances for various bacteria. The experiment was performed in Omnilog PM automatic system using GEN III MicroPlate™ to test the utilization of 71 carbon sources. To check whether the model can get the result consistent with the experiment, the biomass reaction in the model was set as the objective equation to calculate the FBA value, and the tested substance was used as the only exogenous intake.

### 2.5 Modeling with proteome data

The proteome data was integrated into the metabolic network by E-Flux method (Colijn et al., 2009) to construct models of rhizobium in the logarithmic and stable phases in the free-living condition and parasitized in cultivated soybean and wild-type soybean under symbiotic condition. The proteome data was obtained from Rehmen’s work (Rehman et al., 2019), and the average of the peptide values in three replicate experiments was adopted as the protein expression data. Protein expression data was mapped to the model by parsing the gene-protein-reaction (GPR) rules associated with each reaction. Flux variability analysis (FVA) was used to determine the boundary of each metabolic reaction. The lower bound of the reactions that were more than 0 and the upper bound of the reactions that were less than 0 were set to 0. The ultimate boundary was calculated by E-Flux method and the results are in Supplementary Table S2. Since Rehmen’s work only had *S*. *fredii* CCBAU25509 proteome data about wild-type soybean accession W05, we employed the protein homology mapping method (Kellis et al., 2004) to match the *S*. *fredii* CCBAU25509 genes with the *S*. *fredii* CCBAU45436 genes, using “Compare Two Proteomes” tool of KBase (Arkin et al., 2018) with a minimum suboptimal best bidirectional hit (BBH) ratio of 90%. After obtaining the original results, we chose the best matching outcome in the bidirectional comparison results. The original results from KBase and the processed data can be found on GitHub and in Supplementary Table S3.

### 2.6 Analysis of essential genes and reactions

By identifying the essential genes and reactions, we can have deep insights into the life process of living organisms, which will be the key tool to control it freely. The “singleGeneDeletion” function in the COBRA Toolbox was used for the prediction of essential genes, and the “singleRxnDeletion” function was used for the prediction of essential reactions. Among them, genes and reactions of which the ratio of the growth rate of mutants to normals is less than or equal to 0.05 were considered as essential genes and essential reactions. Detailed information on the essential genes and essential reactions can be found in Supplementary Table S4. To ensure the reliability of the essential genes, we used the data from the literature and performed the function of BLASTP with genes in the Database of Essential Genes (DEG) (http://origin.tubic.org/deg/public/index.php/index) (Luo et al., 2021). Moreover, Geptop (http://guolab.whu.edu.cn/geptop/) was used to further predict the essentiality of genes, which was a powerful tool for prediction of essential genes of prokaryotic organisms (Wen et al., 2019). The genes were validated as essential genes if they were reported in literature or had identity score over 50% in BLASTP with genes in DEG or had an essentiality score over 0.24 predicted by Geptop. Detailed information of validated results can be found in Supplementary Table S5.

### 2.7 Analysis tools

Flux balance analysis (FBA) simulates optimal metabolism at steady-state, which is a useful tool for predicting flux distributions in genome-scale metabolic models and models integrated with various omics data (Orth et al., 2010). Flux variability analysis (FVA) can help calculate the range of flux values that can be achieved for each reaction in the model. COBRA Toolbox v3.0 was mainly used for model construction, FBA, FVA analysis, and essentiality analysis of genes and reactions. COBRApy was mainly used to convert sbml format into json format (Ebrahim et al., 2013). Escher (https://escher.github.io/#/) was used for the mapping of metabolic network (King et al., 2015). All simulations were performed in MATLAB (R2023a) and the solver was GLPK.

## 3 Results and discussion

### 3.1 Reconstructed genome-scale metabolic network model *i*AQY970 and comparison with *i*CC541

In this research, based on the previous *i*CC541 model, a new genome-scale metabolic model *i*AQY970 for *S*. *fredii* CCBAU45436 was reconstructed according to the latest gene annotation information from NCBI, which contained 970 genes, 1,052 reactions and 942 metabolites including two cellular phases. The new model replaced old locus tag and updated the previous enzyme annotation information from BRENDA (Chang et al., 2021) database. In addition, the defects of wrong reaction direction and metabolite duplication in the previous model were further corrected, and the gaps were lessened by filling. Consequently, a more complete metabolic network map was drawn and can be found on GitHub. The detailed information of *i*AQY970 model can be found on the website (https://github.com/AnqiangYe/iAQY970/tree/main).

**FIGURE 2:**
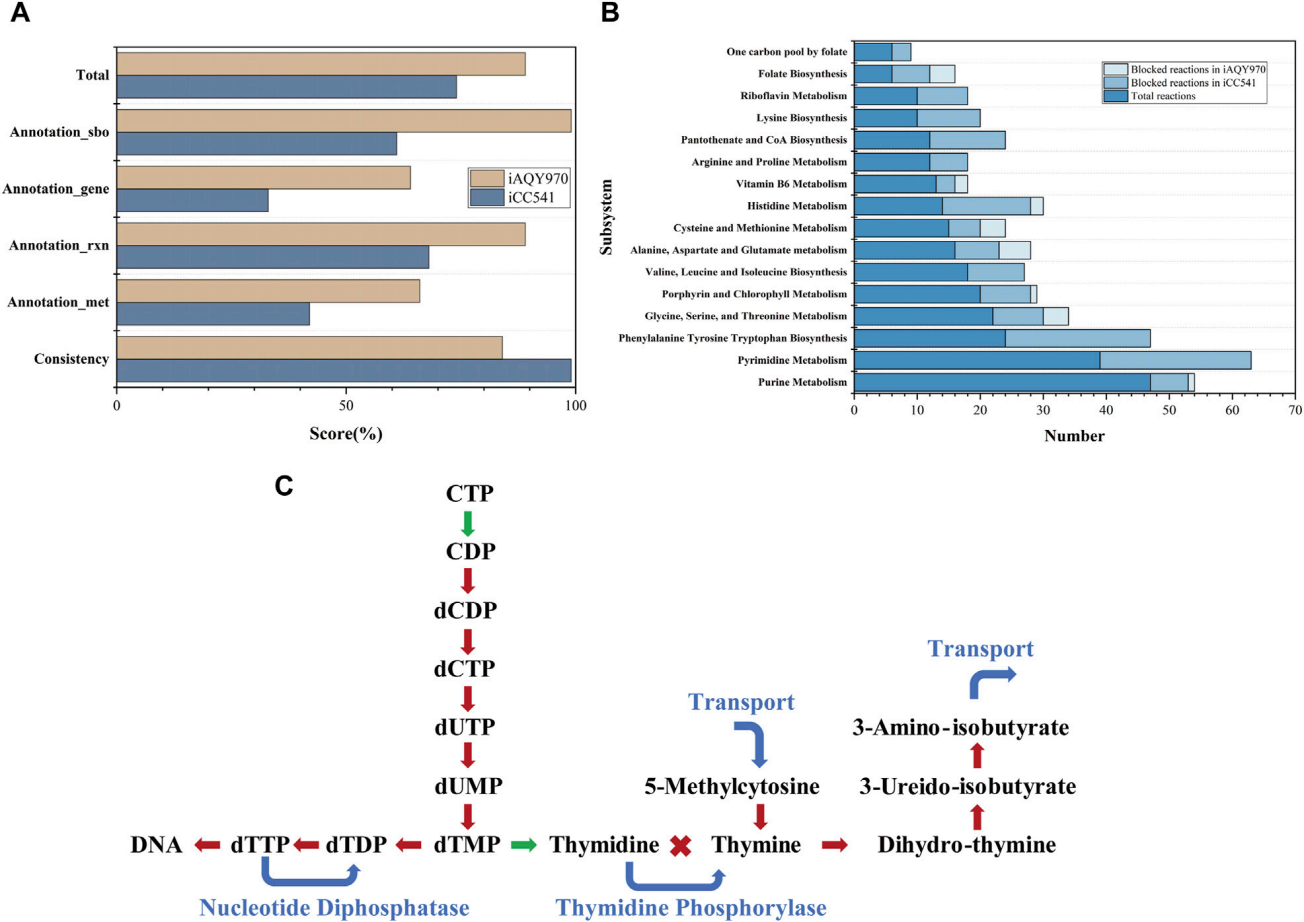
**(A)** The result of MEMOTE test. **(B)** The number of reactions and the number of blocked reactions contained in each subsystem of either model. **(C)** Gap-filling in the pathway of Pyrimidine Metabolism, the red lines represent the blocked pathways in *i*CC541 model, the green lines represent the correct pathways in *i*CC541 model, and the blue lines represent the added pathways in *i*AQY970 model.

The general features of *i*AQY970 and that compared with *i*CC541 are in Table 1. In total, *i*AQY970 contains 970 genes, accounting for 14.7% of the whole genes, while *i*CC541 contains 541 genes, accounting for only 8.2%. The model *i*AQY970 has higher gene coverage conducive to the more comprehensive simulation of the metabolic process. Moreover, *i*AQY970 can simulate the metabolic process both in free-living and symbiotic states but *i*CC541 considered only a symbiotic state. The two models were evaluated using a standardized genome-scale metabolic model test suite named MEMOTE which can assess model consistency including stoichiometric consistency, mass balance, charge balance and metabolite connectivity and annotation of metabolite, reaction, gene and SBO. Comparisons of MEMOTE scores of the two models are shown in Figure 2A. The MEMOTE version was 0.16.1 and the full reports of MEMOTE score can be found on GitHub. The scores indicate that *i*AQY970 has more complete annotation than *i*CC541. The number of reactions and the number of blocked reactions contained in each subsystem of either model are shown in Figure 2B. The results show that *i*CC541 has 167 blocked reactions, accounting for 31% of the total, but the new model has 269 blocked reactions, accounting for 25.6% of the total. The total number of reactions increased, but the ratio of blocked reactions decreased. Meanwhile, some blocking reactions in the old model were repaired by the construction of the new model.

**TABLE 1:** General features of *i*AQY970 and compared with *i*CC541.

Model features	*i*AQY970	*i*CC541
Total genes	6,577	6,577
Genes associated in model	970	541
Gene coverage	14.7%	8.2%
Total reactions	1,052	538
Enzymatic reactions	885	481
Transport reactions	89	24
Exchange reactions	54	23
Demand reactions	28	1
Gene-associated reactions	850	489
Not gene-associated reactions	202	49
Spontaneous reactions	8	8
Metabolites	942	508
Blocked reactions^a^	269	167
Blocked reactions ratio	25.6%	30.8%
MEMOTE score	89	70
Simulation state	Free-living, symbiosis	Symbiosis

^a^ Blocked reactions exclude exchange reactions and transport reactions.

According to the blocked reactions, we repaired the gaps in *i*CC541. The 167 blocked reactions in *i*CC541 were lessened to 39. In the pathway of Pyrimidine Metabolism, the pathway from 5-methylcytosine to 3-amino-isobutyrate was blocked for lack of sink reactions and demand reactions, which were filled by transport reactions. No reaction can connect thymidine and thymine, so we added reaction of phosphate deoxy-alpha-D-ribosyltransferase to connect the two metabolites. Since the rhizobium had nucleotide diphosphatase (EC 3.6.1.9), we added reaction of dTTP diphosphohydrolase to make the flux of dTTP circulate. The process is shown in Figure 2C.

In symbiosis condition, the rhizobium used the malate and succinate from host plant as carbon source to keep flux work and produce symbiotic products in return. The content of the symbiotic product was obtained from *i*CC541, and the uptake of malate and succinate were set to 1.44 mmol g^−1^ h^−1^ and 1.38 mmol g^−1^ h^−1^, respectively according to literature (Contador et al., 2020). FBA analysis was performed in the symbiotic case, and the flux of symbiotic product showed that *i*CC541 was 0.0487 mmol/gDW/h and *i*AQY970 reached 0.9528 mmol/gDW/h (Table 2). Also, the flux of energy and reducing factors were faster in *i*AQY970. We found that *i*CC541 cannot completely reach the maximum uptake limitation of malate and succinate which may be one important reason for the low flux of symbiotic products. Moreover, due to the addition of sink reaction the flux of symbiotic products improved.

**TABLE 2:** Comparison of symbiosis yield and production of energy and reducing factors.

Model	NADH/NADPH production rate (mmol/gDW/h)	ATP production rate (mmol/gDW/h)	Symbiotic production rate (mmol/gDW/h)	Succinate uptake rate (mmol/gDW/h)	Malate uptake rate (mmol/gDW/h)
*i*CC541	5.32	1.05	0.0487	0	0.362
*i*AQY970	29.63	29.37	0.9528	1.38	1.44

### 3.2 Phenotyping data analysis

**FIGURE 3:**
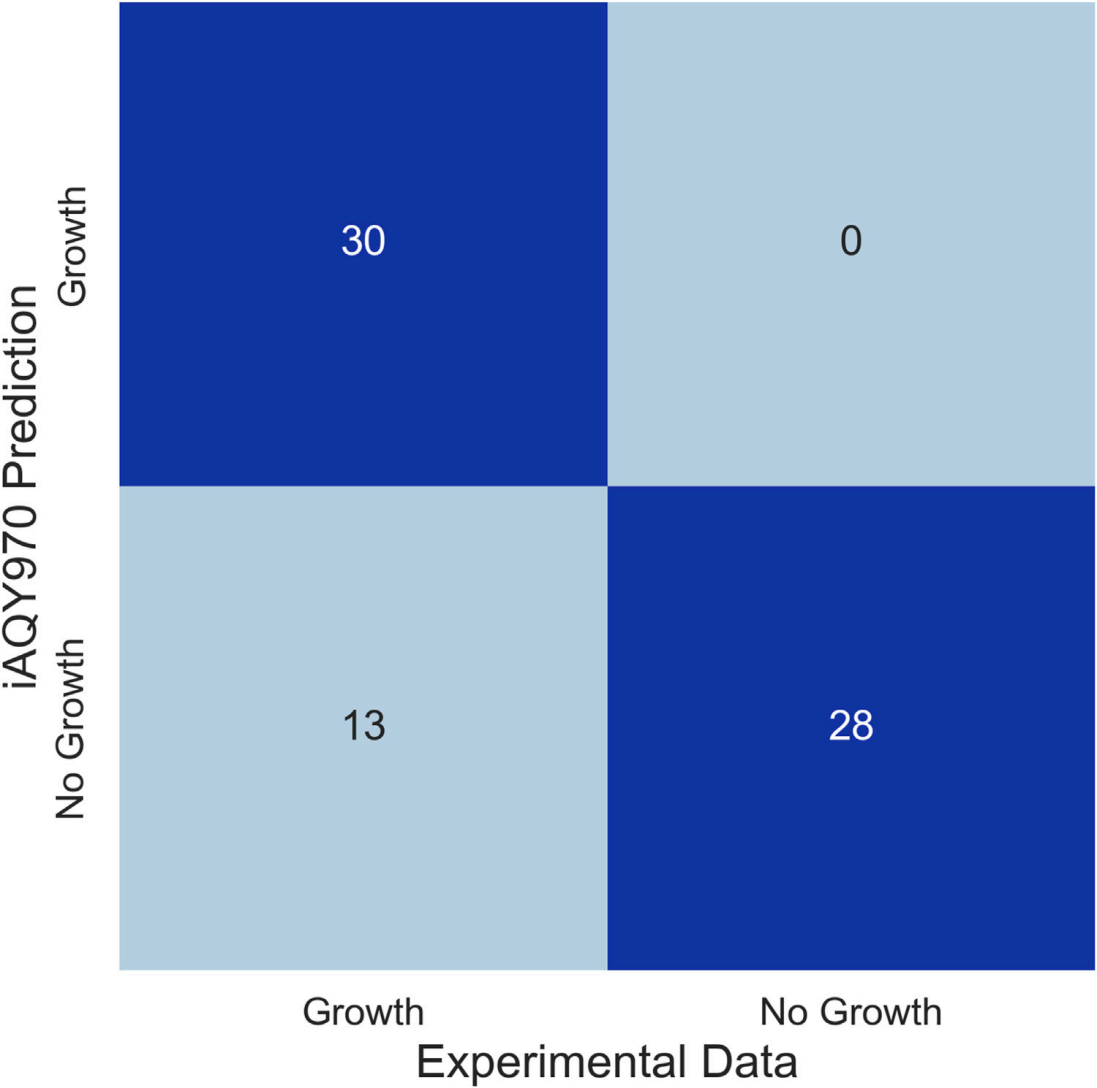
Comparison of the results between *i*AQY970 prediction and Biolog phenotype microarray.

Biolog phenotype microarray was performed to validate the capacity of the model reflecting the utilization of different carbon sources. There were 43 carbon sources that can be used by *S*. *fredii* CCBAU45436 among 71 carbon sources. To compare the model and experiment result, we made each substance as the only intake carbon source and calculated the flux by FBA analysis using a minimal medium. The results of simulation in differing from the experiment were used to modify the model reconstruction. Since we firstly only took succinate and malate into account, we added more exchange and transport reactions to ensure the model can intake the other carbon sources and gap-filling reactions were added to connect the carbon sources with the metabolites in the model. Finally, among 43 carbon sources can be used by *S*. *fredii* CCBAU45436 in the experiment, 30 of them can be utilized for simulation. Among the 13 carbon sources inconsistent with the experiment, 8 of them (Gentiobiose, D-Turanose, β-Methyl-D-Glucoside, D-Fucose, D-Glucuronic Acid, Glucuronamide, Methyl Pyruvate, β-Hydroxy-D, L Butyric Acid) were lack of information in databases like KEGG or ModelSEED indicating existing limitation in model reconstruction. Due to little knowledge of these carbon sources, it was hard to contain them into the genome-scale metabolic network model. After model modification, among 71 carbon sources, 58 carbon sources were consistent with the experiments (Figure 3) which showed high accuracy of *i*AQY970 model (81.7%). Detailed results of the experiment can be found in Supplementary Table S6.

### 3.3 Analysis of proteome data combined with metabolic network models

In order to further analyze the accuracy of the model simulation in free-living and symbiotic states, proteome data analysis was used to verify the accuracy of the model. With the increasing data of multi-omics, it was popular to build a constraint-based metabolic model based on GSMM using transcriptome, proteome and thermodynamic data which can reflect specific conditions. Using proteome data instead of transcriptome data was a good method to avoid the deviation between actual protein expression and transcriptional level expression.

The proteome data were from Rehmen’s work (Rehman et al., 2019), and the number of peptides was adopted as the protein expression profile. The specific models were built from *i*AQY970 by E-Flux method. The number of proteins expressed by each gene was used to determine the lower bound and upper bound of every reaction. The distribution of agent flux in the logarithmic and stable phases and their maps can be found on GitHub (/Map), and the metabolic flux and its upper and lower bounds are shown in the Supplementary Table S7. The final biomass synthesis flux in the logarithmic phase was 0.4009 mmol/gDW/h and in the stable phase was 0.3340 mmol/gDW/h (Table 3), indicating that in the logarithmic phase the biomass synthesis was faster than in the stable phase, which was consistent with the fact that bacteria grew faster in logarithmic phase and accumulated more biomass during this period.

**TABLE 3:** Flux of objective reactions using proteome data.

State	Condition	Biomass production rate (mmol/gDW/h)	Symbiotic production rate (mmol/gDW/h)
Free-living	logarithmic phase	0.4009	-
Free-living	stable phase	0.3340	-
Symbiosis	W05	-	0.5720
Symbiosis	C08	-	0.9528

Since there was currently only *S. fredii* CCBAU25509 proteome data on wild soybean accession W05 in symbiotic case, we used the protein homology matching method to map the gene expression of the *S. fredii* CCBAU45436 to the expression data of the *S. fredii* CCBAU25509. We selected the overlap with the highest hit rate in the genomes of the two species, and the mapping and hit rates of the two species were shown on GitHub. Similarly, we used the proteome data to build models under two conditions. The fluxes of each metabolic reaction in cultivated soybean and wild soybean can be found in Supplementary Table S7. In the case of symbiosis, the symbiotic reaction flux was 0.9528 mmol/gDW/h in cultivated soybean and 0.5720 mmol/gDW/h in wild soybean (Table 3), indicating that the nitrogen-fixing activity of nitrogen-fixing bacteria was better in cultivated soybean, which was consistent with the actual situation. When parasitized in wild soybean, the rhizobia had more flux in fatty acid metabolism but the uptake of succinate reduced to zero indicating poor capacity of utilizing of carbon sources from host plant which might be the reason for less flux of symbiotic reaction.

### 3.4 Analysis of essential genes and essential reactions

According to the gene-protein-reaction (GPR) association, genes were classified as essential genes and non-essential genes determined by whether the reactions should carry nonzero flux to satisfy the objective equation. The prediction of essential genes in a free-living state showed that a total of 143 genes would affect growth, of which 78 were essential genes. The prediction of essential genes in the symbiotic state showed that a total of 184 genes would affect growth, of which 94 were essential genes.

**FIGURE 4:**
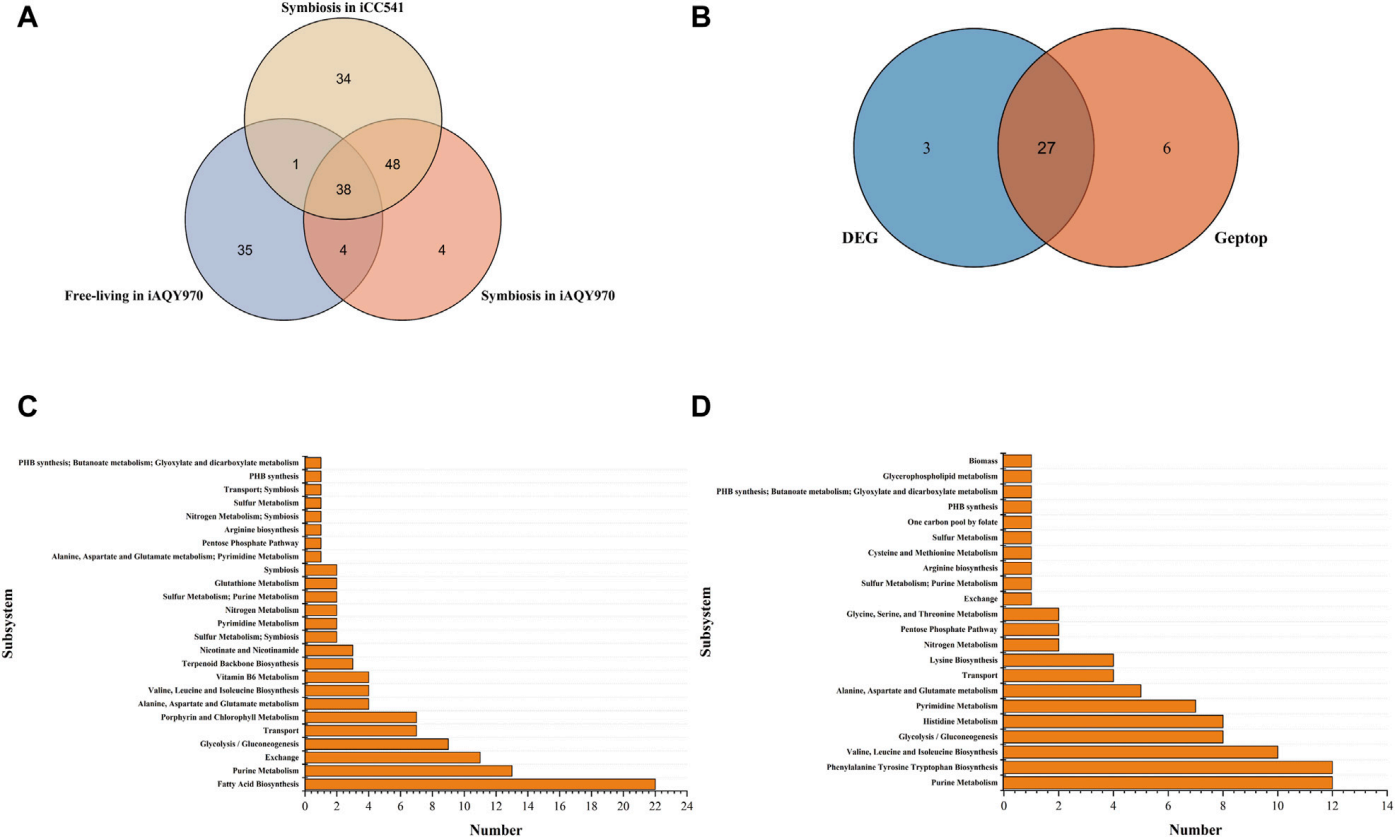
**(A)** The Venn graph of predicted essential genes in *i*AQY970 and *i*CC541 model. **(B)** Essential genes predicted by DEG and Geptop in symbiotic condition. **(C)** Essential reactions predicted in *i*AQY970 model under symbiotic condition. **(D)** Essential reactions predicted in *i*AQY970 model under free-living condition.

The Venn graph of predicted essential genes in *i*AQY970 and *i*CC541 model was shown in Figure 4A. There were 86 genes predicted both by *i*AQY970 and *i*CC541 in symbiotic condition, indicating great consistency in two models. As many researches involved data of symbiotic genes, they were used to verify our prediction. Among the essential genes, 44 genes were confirmed by literature information (Supplementary Table S5), and 30 genes were confirmed by BLASTP against Database of Essential Genes (DEG) (Supplementary Table S5), which determined essential genes when identity score was more than 50%. In addition, 33 genes were predicted as essential genes by Geptop, and the comparison of essential genes predicted by DEG and Geptop was shown in Figure 4B. In total, 27 genes were predicted as essential genes both by DEG and Geptop which showed good consistency of their outputs. Among all essential genes predicted by *i*AQY970, 74 genes were confirmed to be right based on the third part proof, retaining 18 genes without proof and 2 were contradictory with the literature. *Nif* genes played crucial role during SNF by encoding the nitrogenase complex and regulatory proteins involved in nitrogen fixation (Lindstrom and Mousavi, 2020). In *i*AQY970, three *nif* genes, AB395_RS31805, AB395_RS31800 and AB395_RS31060 were added firstly and it was supposed to cause higher efficiency in nitrogen fixation after such supplement. In this model, genes in the TCA cycle, AB395_RS29400 and AB395_RS18105 (acetyl-CoA carboxylase, EC 6.4.1.2), were classified as essential genes indicating that the TCA cycle had a great impact on energy which was important during nitrogen fixation. In previous report, the lack of *ilv* genes might result in no nodule formation which destroyed nitrogen fixation (Prell et al., 2009) so that not only *ilv*D (AB395_RS15445) but also *ilv*N (AB395_RS10030) was added in our model.

As Figure 4A showed, there were 42 essential genes both in free-living and symbiotic condition, which indicated their great importance during whole life of the rhizobium. For example, although it was reported that PHB was necessary during the symbiotic period (Udvardi and Poole, 2013), it was still vital in free-living condition to keep the organism alive. In addition, *pur* family genes also had great importance in the life of the rhizobium, as *pur*L (AB395_RS08020), *pur*Q (AB395_RS08000), *pur*M (AB395_RS04210), *pur*D (AB395_RS02495), *pur*S (AB395_RS07990), *pur*H (AB395_RS17830) appeared in result and were checked to be right. Genes of *pur* family mainly affected the synthesis of phosphoribosylformylglycinamidine which was the middle compound to connect Purine Metabolism with Thiamine Metabolism and Alanine, Aspartate and Glutamate metabolism.

Essential reactions were predicted to appear particularly in different conditions. The essential reactions in different subsystems were shown in Figures 4C, D. Fatty Acid Biosynthesis showed quite difference in two conditions, which was important in symbiotic condition but weighed little in free-living condition. Acyl carrier protein was prominent in forming symbiotic product but seemed not necessary in free-living condition. In contrast, Phenylalanine Tyrosine Tryptophan Biosynthesis occupied an important position in free-living condition, indicating adequate demand for aromatic amino acids. Moreover, Purine Metabolism and Glycolysis/Gluconeogenesis were crucial in the life of the rhizobium, and during symbiotic period it showed more active phenomenon in exchange reaction.

### 3.5 Modules of nitrogen fixation

Rhizobia have the capacity of biological nitrogen fixation in the symbiotic state. However, since there were too many genes and reactions during nitrogen fixation, it was difficult to find core modules for nitrogen fixation. In order to mine the nitrogen fixation module, we analyzed four possible nitrogen fixation modules based on the new model according to the production of reductive ferredoxin because nitrogenase (EC 1.18.6.1) was the top priority enzyme in nitrogen fixation and it needs reductive ferredoxin as the substrate. The reactions (Ferredoxin NADP reductase, Isopentenyl-diphosphate, Carbon monoxide dehydrogenase and Hydrogen-sulfide) can produce reductive ferredoxin (Table 4), and we set the lower bound and upper bound of one reaction to 0 successively and calculate the NADH/NADPH production, ATP production, Symbiotic production and Symbiotic nitrogen fixation which can be found in Table 5.

**TABLE 4:** Reactions which can produce reductive ferredoxin.

Reaction	Enzyme	EC number	Equation
Ferredoxin NADP reductase	Ferredoxin-NADP + reductase	1.18.1.2	h + nadp +2 fdxrd <=> nadph +2 fdxox
Isopentenyl-diphosphate	Xanthine dehydrogenase	1.17.1.4	2 h + 2 fdxrd + h2mb4p <=> h2o + 2 fdxox + ipdp
Carbon monoxide dehydrogenase	-	-	h2o + co + 2 fdxox <=> co2 + 2 h + 2 fdxrd
Hydrogen-sulfide	Sulfite reductase	1.8.7.1	h2s + 6 fdxox +3 h2o <=> so3 + 6 fdxrd +7 h

Note: h: H+; fdxrd: Reductive ferredoxin; fdxox: Oxidized ferredoxin; h2mb4p: 1-hydroxy-2-methyl-2-(E)-butenyl 4-diphosphate; ipdp: Isopentenyl diphosphate; co: CO; co2: CO2; h2s: Sulfide; so3: Sulfite.

**TABLE 5:** Comparison of nitrogen fixation in four modules.

Module	NADH/NADPH production rate (mmol/gDW/h)	ATP production rate (mmol/gDW/h)	Symbiotic production rate (mmol/gDW/h)	Symbiotic nitrogen fixation rate (mmol/gDW/h)
Module 1	28.34	29.89	0.9528	0.3891
Module 2	29.80	29.67	0.9528	0.3891
Module 3	14.86	15.78	0.2745	0.1121
Module 4	29.91	29.89	0.9528	0.3891

Note: Module 1: Nicotinate and Nicotinamide; Module 2: Terpenoid Backbone Biosynthesis; Module 3: Carbon Metabolism; Module 4: Sulfur Metabolism.

There were four modules that mainly affected the production of reductive ferredoxin and subsequently influenced the function of nitrogenase: Module 1 (Nicotinate and Nicotinamide), Module 2 (Terpenoid Backbone Biosynthesis), Module 3 (Carbon Metabolism), Module 4 (Sulfur Metabolism). The fluxes of the four nitrogen fixation pathways can be found on GitHub (https://github.com/AnqiangYe/iAQY970/tree/main/Map/Flux). The symbiotic production flux of Module 1, Module 2 and Module 4 were 0.9528, and the symbiotic production flux of Module 3 was 0.2745. According to the results, Module 3 had defect in nitrogen fixation and the reason had to do with the energy.

To further find the key genes which can enhance the production of Fixed NH3, we adopted the minimization of metabolic adjustment (MOMA) algorithm (Segre et al., 2002) to identify potential genes (Table 6). The upper bound of Fixed NH3 exchange reaction was set to 0.001 mmol/gDW/h and reactions were increased by FBA simulation in each module. By overexpressing genes, ferredoxin NADP reductase (EC 1.18.1.2), xanthine dehydrogenase (EC 1.17.1.4) and sulfite reductase (EC 1.8.7.1) directly improve the production of reductive ferredoxin, of which sulfite reductase (EC 1.8.7.1) had the most significant impact on the production of Fixed NH3. In module 2, the overexpression of 4-hydroxy-3-methylbut-2-en-1-yl diphosphate reductase (EC 1.17.7.4) and isopentenyl-diphosphate DELTA-isomerase (EC 5.3.3.2) can increase the production of Isopentenyl diphosphate and Dimethylallyl diphosphate in Terpenoid Backbone Biosynthesis, which indirectly improves the production of reductive ferredoxin by the reaction of Isopentenyl-diphosphate. However, in simulation overexpression of Carbon monoxide dehydrogenase did not work and the entire metabolic network collapsed due to the inability to reach the overexpression value, resulting in a calculated result of 0. The enzymes sulfite reductase (EC 1.8.7.1) and nitrogenase (EC 1.18.6.1) were excellent target genes for enhancing nitrogen fixation since their overexpression led to huge improvement in simulation. According to the simulation, we should not only overexpress the nitrogenase (EC 1.18.6.1) directly but also improve the Sulfur Metabolism and Terpenoid Backbone Biosynthesis as well as silencing the genes that can consume reductive ferredoxin.

**TABLE 6:** Target genes overexpressed by MOMA analysis and the predicted metabolism situation.

Reaction^a^	Enzyme	EC number	Fixed NH3 exchange rate (mmol/gDW/h)	Symbiotic production rate (mmol/gDW/h)	Wild type Fixed NH3 exchange rate (mmol/gDW/h)	Wild type symbiotic production rate (mmol/gDW/h)	Module^b^
rxn05937	Ferredoxin-NADP + reductase	1.18.1.2	3.7941	0.0012	0.001	0.0012	Module 1
rxn04113	Xanthine dehydrogenase	1.17.1.4	3.7941	0.0012	0.001	0.0012	Module 2
rxn08352	4-hydroxy-3-methylbut-2-en-1-yl diphosphate reductase	1.17.7.4	2.5881	0.0012	0.001	0.0012	Module 2
rxn00830	Isopentenyl-diphosphate delta3-delta2-isomerase	5.3.3.2	3.7940	0.0012	0.001	0.0012	Module 2
rxn05902	Sulfite reductase	1.8.7.1	13.7941	0.0012	0.001	0.0012	Module 4
rxn06874	Nitrogenase	1.18.6.1	20	0.0012	0.001	0.0012	Module 1
							Module 2
							Module 4

^a^ ModelSEED ID., Rxn05937: ferredoxin NADP, reductase; rxn04113: isopentenyl-diphosphate; rxn08352: 1-hydroxy-2-methyl-2-(E)-butenyl 4-diphosphate reductase; rxn00830: isopentenyl-diphosphate delta3-delta2-isomerase; rxn05902: hydrogen-sulfide; rxn06874: nitrogenase.

^b^ Module 1: Nicotinate and Nicotinamide; Module 2: Terpenoid Backbone Biosynthesis; Module 4: Sulfur Metabolism.

## 4 Conclusion

In general, we updated the metabolic network based on the first-generation *i*CC541 network model through a manual scheme, and designed the new model *i*AQY970. Compared with the previous model, *i*AQY970 had higher gene coverage and reflected a more complete metabolic process of *S. fredii* CCBAU45436. By integrating the proteome data, the metabolic network models under two specific conditions were constructed, and the results showed that the logarithmic phase of the metamorphosis was higher than the stable phase in the free-living case. And the nitrogen fixation reaction flux of parasitism in cultivated soybean was higher than that in wild soybean in the symbiotic case. These simulation results were consistent with the actual situation, which further verified the reliability of the metabolic network. Moreover, 94 essential genes were predicted by *i*AQY970, which were evaluated also by DEG and Geptop. Finally, four key nitrogen fixation modules were analyzed and some target genes were predicted by MOMA according to the *i*AQY970 model, which provided helpful guidance for the subsequent optimization of the nitrogen fixation process of the rhizobium. This more comprehensive metabolic model can help researchers better understand the metabolic process of the rhizobium and provide a powerful tool for its development and utilization.

## Data Availability

The datasets presented in this study can be found in online repositories. The names of the repository/repositories and accession number(s) can be found in the article/Supplementary Material.
